# Popular Science Department

**Published:** 1882-10

**Authors:** 


### POPULAR SCIENCE DEPARTMENT.

### THE INTERNATIONAL CONGRESS OF HYGIENE AT GENEVA.

From September 4 to 9 last, between five and six hundred sanitarians and physicians were assembled in Congress at Geneva, under the Presidency of the veteran Professor Lombard, who must now be about 80 years old. The magnitude and interest of the meeting was in part due to the fact that owing to the burning of the Sanitary

Exhibition in Berlin there was no meeting of the German Public Health Association this year, and the Congress took its place to a considerable extent.

The first important paper was presented by M. Pasteur, September 5, the subject being the attenuation of virus.

Referring to his previously published results obtained by modifying the specific virus of chicken cholera or splenic fever in such a way that it would produce a slight and temporary disease which would protect against the more deadly forms, he proceeded to give the results of similar attempts with the specific germ which is found in the saliva of certain persons, and which produces a very fatal form of septicæmia, or blood poisoning, in the lower animals, and especially in rabbits. In this, as in the two forms of disease above mentioned, the diminution in the virulence of the poison was effected by successive cultures under the influence of the oxygen of the air.

He then gave a brief account of some experiments made upon a disease which prevailed epidemically in Paris in 1881, and which was known as the typhoid fever of horses. This was found to be due to a minute organism which could be inoculated in rabbits, producing a very fatal disease strongly resembling typhoid fever. By culture this germ was so far deprived of its specific virulence that instead of causing death it produced only great local inflammation, and acted like vaccine in preventing deadly effects from the unweakened virus subsequently applied. The paper closed with a sharp reply to the criticisms upon his previous work by Koch in vol. 1 of the publication of the Imperial German Board of Health. To this Koch, who was present, replied briefly, denying that there was anything new in Pasteur's paper, or that it had any special connection with hygiene, and saying that he would reply through the press. As Pasteur knew no German and Koch no French it was clearly impossible to continue the discussion to any good purpose.

The general session of September 6 was occupied with a discussion on the contagion of phthisis (or consumption) based on a report presented by Prof. Corradi, of Pavia. His conclusions were that under some conditions, as yet little understood, a consumptive patient is a source of danger to those closely associated with him, and that it is prudent to abstain from the meat and milk of tuberculous animals.

Dr. Leudet, of Rouen, gave some interesting statistics on this question, indicating the probability of the transmission of the disease from husband to wife, but also showing that the disease is often transmitted to the children without affecting the mother. The general conclusion was that the subject is very obscure, and that positive evidence is still wanting.

In the general session of September 8, Prof. Lombard stated his conclusions with regard to the effects of altitude in a hygienic and therapeutic point of view.

The effect of great altitude is to produce distress owing to insufficient supply of oxygen. Modern altitudes tend to produce frequent and deep inspirations, to increase the capacity of the chest, and to stimulate all the functions of the body. They also tend to prevent and to cure consumption. Prof. Paul Bert, of Paris, who has made many and valuable investigations as to the effect of increased and diminished pressures of the atmosphere on living beings, gave an interesting account of some of his experiments. He found that in animals which live at great altitudes the blood had a greater capacity for absorption of oxygen than the blood of the same animal when habituated to a lower level—and the whole of his researches went to prove that it is the diminished oxygen which produces the effects of altitude.

The Congress was eminently French in character, and so was the sanitary exhibit which accompanied the meeting. The Germans are reserving themselves for the great exhibition and meeting at Berlin next year.—*Sanitary Engineer.*

### DR. BARKER ON SEWER-GAS AND DISEASE.

The special poisons to which I now refer are the gases resulting from defective plumbing, to which all classes—the poor occupants of tenement-houses, those who are able to command the necessaries and many of the luxuries of life, and those who live in the most expensive houses, and whose riches can buy everything but health—are like exposed. None but physicians can know how general this poison is, and how positively it explains much of the disease that they are called upon to treat, and of the many sad deaths which follow.

When I assert that it is a daily experience with me to see persons whose general health is suffering from this poison, as manifested by *malaise*, loss of appetite and strength, slight febrile symptoms, diarrhœa, physical and mental depression ; and that I have seen infants, children and adults suffering from diptheria, scarlet fever of a mild type, complicated with this disease and destroying life; those in vigorous health, students in colleges, ambitious and active young men in the professions or in the commercial or financial world, stricken down by typhoid fever, some struggling through the disease and others dying ; and that the cause has been demonstrated to be this poison—I only state facts which are common in the experience of all physicians in this city. In some cases this has been the result of ignorance of the very unsanitary conditions which environed them. For example, two young men were stricken down with typhoid fever, one of whom died. They were not acquaintances, but occupied offices in the same building, in the vicinity of Wall Street. On investigation. it was found that there was not a trap in the whole building. In a house in which, but a few months before, several hundred dollars had been expended to put the building in perfect condition, a young man died of typhoid fever, and others of the family became ill, when it was found that a defective waste-pipe was saturating the house with poisonous gas. But such facts as these are so common and so well known to the profession that I need not dwell upon them.—*From article by* DR. FRANK H. HAMILTON, *on* "*Sewer-Gas,*" *in Popular Science Monthly for November.*

### A POCKET PHOTOMETER.

Mr. Robert Sabine, the well-known electrician, has devised a handy little pocket photometer, which will be useful for estimating, approximately, the intensity of different electric lights. It is based on Ritchie's method, and consists of a telescopic tube, having at one end a square box, two opposite sides of whlch are glazed with squares of opal glass, cut from the same sheet. Within this box is a prism, or angle of a prism, made up of two plane mirrors, meeting each other at a right angle, the apex of which points up the telescopic draw tube. This tube is fitted with an eye piece at the other end, and a small lens, so as to get a good focus, in order to see the mirrors plainly. The principle of Ritchie's photometer consists in comparing the intenities of the two lights, as reflected on the mirrors or white card surfaces ; and the observer moves until the relative distances between himself and the lights give a sensibly equal illumination of the mirrors. These distances then give the relative intensities of the lights by the law of inverse squares. In Mr. Sabine's photometer, the lens and draw tube take the place of the small hole employed by Ritchie, and this requires the tube to be drawn out about four inches. Were it not for the lens, the tube would require to be much longer in order to get a good view of the mirrors.—*Engineering.*

### A NEW PROFESSION FOR WOMEN.

---

The stranger in New York who may chance to visit the east side of the city in the neighborhood of Twenty-sixth street, will have his attention called to a long, grayish, four-story prison-like structure, with a wing, situated in a block which extends to the East River, and inclosed by a high, forbidding stone wall. This is Bellevue Hospital, the chief free public institution of the kind in New York. For many years it has been famous for the high medical and surgical skill of which it is the theatre, its faculty embracing many leading members of the profession in the city. For many years to come it is likely to be popularly associated with another high development of the curative arts,—the results of the founding, in 1873, of the Bellevue Training-school for Nurses, and a new profession for women in America. * * *

To find a person capable of taking charge of such an institution proved a difficult task. Miss Bowden, otherwise known as "Sister Helen, of All Saints," then of Baltimore, but formerly of the well-known school at University College, London, was finally selected. Equal difficulty was experienced in procuring assistants for her. Advertisements were inserted in the journals, and physicians were applied to ; but such was the scarcity of edueated nurses in this country at that time, that, after a search of many months, and after the most liberal offers, ouly four were found who were in any wise capable, one of whom proved inefficient. Later on, Sister Helen, compelled to return to England, was succeeded by Miss Perkins, of Norwich, Conn., under whose management the school has continued to increase in numbers and usefulness. At first but six pupils were obtained. The scheme adopted—that developed by Miss Nightingale—demanded in the applicant a combination of requisites the mere enumeration of which appalled many who had been encouraged to seek admission to the school. These are : Good education, strong constitution, freedom from physical defects, including those of sight and hearing, and unexceptionable references. The The course of training consists in dressing wounds, applying fomentations, batning and care of helpless patients, making beds, and managing positions. Then follow the preparation and application of bandages, making rollers and linings of splints. The nurse must also learn how to prepare, cook, and serve delicacies for the invalid. Instruction is given in the best practical methods of supplying fresh air, and of warming and ventilating the sick-room. In order to remain through the two years' course and obtain a diploma, still more is required, viz, Exemplaiy deportment, patience, industry and obedience. The first year's experience was far from satisfactory. Among seventy-three applicants, hailing from the various States, only twenty-nine were found that gave promise of ability to fulfill the conditions. Of these, ten were dismissed for various causes before thc expiration of the first nine months. To serve medicine to the patients in the wards of a great public hospital smacks not a little of novelty and of romance, and goes far, at first, to compensate for a hospital's unpleasant surroundings and its odor of disinfectants ; but a short period of wound-dressing and night-watching is sufficient to dispel such illusions. Every year, young women whose abilities warranted their admittance at the commencement of the course, have been permitted to depart before its completion, owing to an evident distaste on their part for the duties imposed upon them. But the managers, though surprised at the result of their first efforts, were not discouraged. As time went by, the number of applicants increased, and though the high standard first established was not departed from, the proportion of those capable of fulfiling the requirments multiplied. Some applicants, who did not seem especially adapted to the work, proved most efficient, and on this topic the managers say that, after their long experience, they have found that the fitness of an applicant can be determined only by absolute trial.

The nurses at the Bellevue school may be divided into two classes ; those who study the art of nursing with a view to gaining a livelihood or supporting their families, and those who look forward to a life of usefulness among the poor sick. All are lodged and boarded free of charge during the two years' course, and are paid a small sum monthly while in the school, to defray their actual necessary expenses, and in order to avoid all distinction between rich and poor, every nurse is expected to receive this pay.

The "Nurses' Home," the headquarters of this school, is No. 426 East Twenty-sixth, a largn and handsome building, erected for the purpose and given to the school by Mrs. W. H. Osborn.—*Century Magazine.*

